# Prediction models for clustered data: comparison of a random intercept and standard regression model

**DOI:** 10.1186/1471-2288-13-19

**Published:** 2013-02-15

**Authors:** Walter Bouwmeester, Jos WR Twisk, Teus H Kappen, Wilton A van Klei, Karel GM Moons, Yvonne Vergouwe

**Affiliations:** 1 Julius Center for Health Sciences and Primary Care, University Medical Center Utrecht, Str. 6.131, PO Box 85500, Utrecht, GA 3508, The Netherlands; 2 Department of Methodology and Applied Biostatistics, Institute of Health Sciences, Vrije Universiteit, Amsterdam, The Netherlands; 3 Department of Perioperative Care and Emergency Medicine, University Medical Center Utrecht, Utrecht, The Netherlands

**Keywords:** Logistic regression analysis, Prediction model with random intercept, Validation

## Abstract

**Background:**

When study data are clustered, standard regression analysis is considered inappropriate and analytical techniques for clustered data need to be used. For prediction research in which the interest of predictor effects is on the patient level, random effect regression models are probably preferred over standard regression analysis. It is well known that the random effect parameter estimates and the standard logistic regression parameter estimates are different. Here, we compared random effect and standard logistic regression models for their ability to provide accurate predictions.

**Methods:**

Using an empirical study on 1642 surgical patients at risk of postoperative nausea and vomiting, who were treated by one of 19 anesthesiologists (clusters), we developed prognostic models either with standard or random intercept logistic regression. External validity of these models was assessed in new patients from other anesthesiologists. We supported our results with simulation studies using intra-class correlation coefficients (ICC) of 5%, 15%, or 30%. Standard performance measures and measures adapted for the clustered data structure were estimated.

**Results:**

The model developed with random effect analysis showed better discrimination than the standard approach, if the cluster effects were used for risk prediction (standard c-index of 0.69 versus 0.66). In the external validation set, both models showed similar discrimination (standard c-index 0.68 versus 0.67). The simulation study confirmed these results. For datasets with a high ICC (≥15%), model calibration was only adequate in external subjects, if the used performance measure assumed the same data structure as the model development method: standard calibration measures showed good calibration for the standard developed model, calibration measures adapting the clustered data structure showed good calibration for the prediction model with random intercept.

**Conclusion:**

The models with random intercept discriminate better than the standard model only if the cluster effect is used for predictions. The prediction model with random intercept had good calibration within clusters.

## Background

Many clinical prediction models are being developed. Diagnostic prediction models combine patient characteristics and test results to predict the presence or absence of a certain diagnosis. Prognostic prediction models predict the future occurrence of outcomes [1].

Study data that are used for model development are frequently clustered within e.g. centers or treating physician [2]. For instance, patients treated in a particular center may be more alike compared to patients treated in another center due to differences in treatment policies. As a result, patients treated in the same center are dependent (clustered), rather than independent. Regression techniques that take clustering into account [3-6] are frequently used in cluster randomized trials and in etiologic research with subjects clustered within e.g. neighborhoods or countries. Surprisingly, such regression models were hardly used in research aimed at developing prediction models [2].

Notably from the domains of therapeutic and causal studies, it has been shown that regression methods that take the clustering into account yield different estimates of the regression coefficients than standard regression techniques neglecting the clustered data structure [5,7,8]. However, in the domain of prediction studies, it is yet unknown to what extent regression methods for clustered data need to be used in the presence of clustered data. Two types of models can be used for analyzing clustered data: marginal models and conditional models [9]. Marginal models, such as the Generalized Estimation Equation (GEE) method, adjust for the clustering nature of data and estimate the standard error of the estimated parameters correctly. The interpretation of GEE results is on the higher cluster level and therefore not suitable for predictions in individual patients [10]. Conditional models estimate predictor effects for patients in the specific clusters. Conditioning on cluster is often done with random effects to save degrees of freedom. Thereby, conditional models allow for predictions of outcomes on the lowest clustering level (here, the patient). Accurate predictions are not necessarily achieved with a random effects model (having different regression parameters compared to a standard model), because the random effects are not readily applicable in new data with new clusters and the clustering in the data may be weak resulting in minor differences between the models.

We explore the effect of including a random intercept in a conditional prediction model compared to standard regression. We use empirical and simulated clustered data to assess the performance of the prediction models. We show that model calibration is suboptimal, particularly when applied in new subjects, if clustering is not accounted for in the prediction model development.

## Methods

### Prediction of postoperative nausea and vomiting

A frequently occurring side effect of surgery is postoperative nausea and vomiting (PONV). To prevent or treat PONV, a risk model was developed to predict PONV within 24 hours after surgery [11]. We used a cohort of 1642 consecutive surgical patients (development sample) that were treated in the UMC Utrecht to develop prediction models. Patients were clustered within 19 treating anesthesiologists [12]. Predictors for the occurrence of PONV included gender, age, history of PONV or motion sickness, current smoking, abdominal or middle ear surgery versus other type of surgery and the use of volatile anesthetics during surgery [13]. Data of 1458 patients from the same center were used to study the validity of the prediction models. Patients included in the validation sample were treated by 19 other anesthesiologists than the patients from the development sample.

### Model development and risk calculation

The prediction models included all before mentioned predictors and were fitted with standard logistic regression or with a random intercept logistic regression model (also known as partial multilevel model). The standard model was fitted with a generalized linear model, including a logit link function. The intercept and predictors were included as fixed effects (i.e. not varying by cluster). The random effect models thus included fixed effects for the predictors plus a random intercept for the effects of clusters (anesthesiologists or centers in the simulation study). The random intercept was assumed to be normally distributed with mean zero, and variance σ^2^_u0_[14].

The predicted risks of PONV for individual patients were calculated with the log-odds transformation of the linear predictor. The risk based on the standard logistic regression model was:

$$P\left(Y_{i}=1|X_{i}\right)=\frac{1}{1+\exp\left(-\left[{\hat{\alpha }}_{\mathrm{standard}}+\sum_{m\in 1,\ldots ,6}x_{\mathrm{im}}\cdot {\hat{\beta }}_{\mathrm{standard},m}\right]\;\right)}$$

where P(Y_i_ = 1) is the predicted risk that a patient *i* will get PONV, given patients’ predictor values X. The linear predictor consist of *â*_standard_ which equals the estimated intercept of the standard model, and $\sum x_{\mathrm{im}}\cdot {\hat{\beta }}_{\mathrm{standard},m}$ which is the sumproduct of the six predictor values of patient i and the six regression coefficients.

From the random intercept logistic regression model, predicted risks were calculated in two ways. The first risk calculation was based on only the fixed effects of the random intercept logistic regression model (called marginal risk calculation):

$$P\left(Y_{i}=1|X_{i}\right)=\frac{1}{1+\exp\left(-\left[{\hat{\alpha }}_{\mathrm{RE}}+\sum_{m\in 1,\ldots ,6}x_{\mathrm{im}}\cdot {\hat{\beta }}_{\mathrm{RE},m}\right]\;\right)}$$

where *â*_RE_ equals the fixed intercept and $\sum x_{\mathrm{im}}\cdot {\hat{\beta }}_{\mathrm{RE},m}$

 is the sumproduct of the six predictor values of patient i and the corresponding fixed regression coefficients of the random effects model. However, the cluster effects were not used for the risk calculation [15]. We explicitly studied this risk calculation since cluster effects are unknown for patients in clusters that are not included in the development data.

The second risk calculation used the fixed and random effects of the random intercept logistic regression model (called conditional risk calculation):

$$P\left(Y_{\mathrm{ij}}=1|X_{\mathrm{ij}}\right)=\frac{1}{1+\exp\left(-\left[{\hat{\alpha }}_{\mathrm{RE}}+\sum_{m\in 1,\ldots ,6}x_{\mathrm{im}}\cdot {\hat{\beta }}_{\mathrm{RE},m}+u_{0j}\right]\;\right)}$$

This risk calculation included the same predictor effects as the marginal risk calculation, plus the random intercept u_0j_ (i.e. the effect of anesthesiologist j). This risk calculation cannot be used in new data of patients treated by new anesthesiologists, since the random effect of the new anesthesiologist is unknown.

### Model evaluation

Apparent and test performance of the prediction models were assessed. Apparent performance is the performance of the prediction model in the development data. Test performance was assessed in a cohort of 1458 new patients treated by 19 other anesthesiologists than the patients from the development sample.

The predictive performance of each of the risk calculations was assessed with the concordance index (c-index) [16], the calibration slope and calibration in the large [17,18]. The calibration slope was estimated with standard logistic regression analysis, modeling the outcome of interest as dependent variable and the linear predictor as independent variable. Calibration in the large was assessed as the intercept of a logistic regression model with the linear predictor as offset variable. The ideal values of the calibration in the large and calibration slope are respectively 0 and 1. Since, standard performance measures ignore the clustered data structure, they can be considered as overall measures. To take clustering into account in the model evaluation, we assessed the predictive performance in individual anesthesiologists (within cluster performance). The within cluster c-index was estimated as the average of the c-indices of the clusters, as described by van Oirbeek [19]. Within cluster calibration was assessed with mixed effect models, with random effects for the intercept and linear predictor (calibration slope) or only for the intercept (calibration in the large).

### Simulation study

We generated a source population which included 100 centers. The number of patients per center was Poisson distributed, with a mean and variance varying per center according to the exponential function of a normal distribution (N(5.7, 0.3)). This resulted in a total of 30,556 patients and a median of 301 patients per center (range 155–552). The dichotomous outcome Y was predicted with 3 continuous (X1-X3) and 3 dichotomous variables (X4-X6). The three continuous predictors were independently drawn from a normal distribution, with a mean of 0 and standard deviations of 0.2, 0.4, and 1. The three dichotomous predictors were independently drawn from binomial distributions with incidences 0.2, 0.3, and 0.4. The regression coefficients of all predictors were 1. To introduce clustering of events, we generated a latent random effect from a normal distribution with mean 0 and variance 0.17. This corresponded to an intraclass correlation coefficient (ICC) of 5%, which was calculated as *σ*^2^_u0_/(*σ*^2^_u0_ + ((*π* ^ 2)/3). The σ^2^_u0_ equals the second level variance estimated with a random intercept logistic regression model [6]. Based on the six predictors and the latent random effect, the linear predictor lp was calculated for each patient. The linear predictor was normally distributed with mean −1.06 and standard deviation 1.41. The linear predictor was transformed to probabilities for the outcome using the formula P(Y) = 1/(1 + exp(−lp)). The outcome value Y (1 or 0) was then generated by comparing P(Y) with an independently generated variable u having a uniform distribution from 0 to 1. We used the rule Y = 1 if P(Y) ≤ u, and Y = 0 otherwise. The incidence of the outcome (P(Y = 1)) was 30% in all source populations, except for the situation with low number of events (incidence = 3%). Further, we varied several parameters in the source population as described above. We studied ICC values of 5%, 15% and 30%; Pearson correlation coefficient values between predictor X1 and the random intercept were 0.0 or 0.4.

Study samples were drawn according to the practice of data collection in a multicenter setting [20,21]. We randomly drew study samples from the source population in two stages. First we sampled 20 centers, and then we sampled in total 1000 patients from the included centers (two-stage sampling). We also studied the performance in study samples with 5 or 50 centers (including respectively 100 and 1000 patients in total). Standard and random intercept logistic regression models were fitted in the study sample, and evaluated in that study sample (apparent performance) and in the whole source population (test performance). The whole process (sampling from source population, model development and evaluation) was repeated 100 times.

Calculations were performed with R version 2.11.1 [22]. We used the lmer function from the lme4 library to perform mixed effect regression analyses [23]. The lrm function of the Design package was used to fit the standard model and estimate overall performance measures [24].

## Results

### Prediction of postoperative nausea and vomiting

The incidence of PONV was 37.5% (616/1642) in the development cohort (Table 1). 19 anesthesiologists treated on average 82 surgical patients (median 64, range 1–460). The incidence of PONV per anesthesiologist varied (interquartile range 29% – 47%), with an ICC of 3.2%. The corresponding variance of the random intercept (0.15) was significantly different from 0 (confidence interval 0.07 – 0.33).

**Table 1:** **Distribution of predictor values and outcome**, **and predictor effects in multivariable logistic regression models**

	**Distribution (N = 1642)**	Interquartile range per anesthesiologist *	**Standard model Beta (95% CI) †**	**Random intercept model Beta (95% CI) †**
*Fixed effect*				
Female gender	882 (54%)	46%-63%	0.75 (0.53 – 0.97)	0.78 (0.54 – 1.01)
Age, years ‡	49 (16.5)	46-51	-0.008 (-0.014 – -0.001)	-0.009 (-0.016 – -0.003)
History of PONV or motion sickness	530 (32%)	30%-36%	0.41 (0.19 – 0.64)	0.42 (0.18 – 0.65)
Current smoking	510 (31%)	20%-33%	-0.43 (-0.66 – -0.19)	-0.45 (-0.69 – -0.20)
Abdominal or middle ear surgery	209 (13%)	4%-16%	0.62 (0.32 – 0.93)	0.57 (0.24 – 0.89)
Volatile anesthetics	844 (51%)	33%-74%	-0.03 (-0.24 – 0.18)	0.19 (-0.09 – 0.46)
Intercept	616 (38%) §	29%-47% §	-0.65 (-1.05 – -0.25)	-0.68 (-1.16 – -0.19)
*Random effect*				
Intercept variance ††	-	-	-	0.15 (0.07 – 0.33)

* interquartile ranges of the predictor distributions per anesthesiologist.

† logistic regression coefficients with 95 percent confidence intervals. Intervals of the random intercept model were based on the t-distribution.

‡ mean (standard deviation).

§ number (percentage) of patients with PONV, rather than the intercept is reported.

PONV = post operative nausea and vomiting.

†† non-parametric confidence interval of the random intercept variance was obtained from a cluster bootstrap procedure.

**Figure 1:**
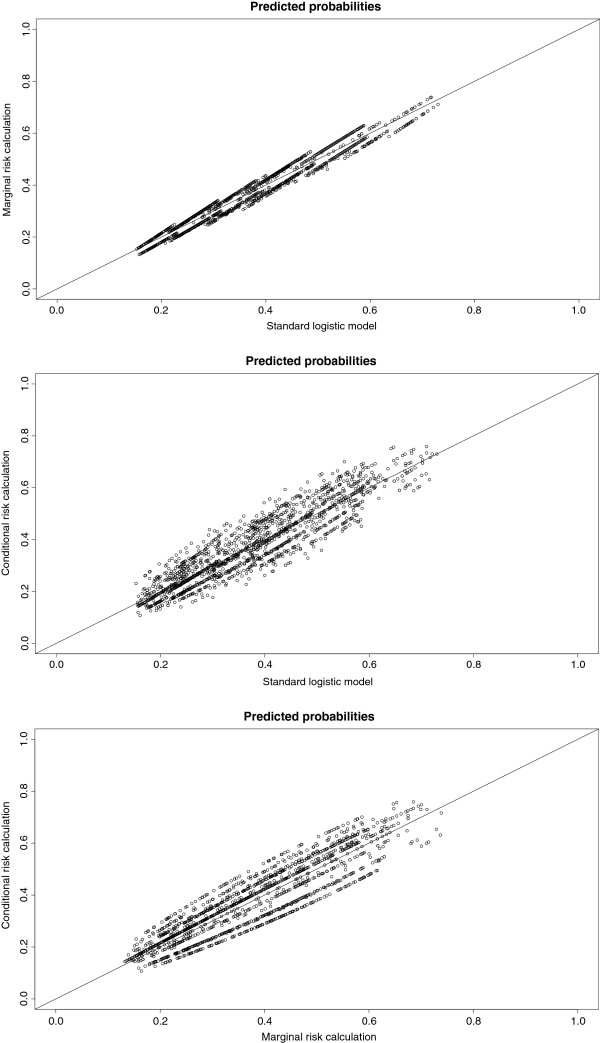
**A**-**C Predicted probabilities from the standard model and from the risk calculations based on the random intercept model.** The predicted risks differed among the models. The diagonal indicates the line of identity (predicted probabilities of the two models are equal).

When the clustering by anesthesiologist was taken into account (random intercept model), the predictive effect of type of anesthetics use (volatile yes/no) was different compared with the standard multivariable model (Table 1). If predictor distributions varied among anesthesiologists, as indicated by a wide interquartile range (Table 1), differences in predictor effects were found between the standard and random intercept model. The variance of the random intercept was 0.15, when the six predictors were included in the model. Consequently, random intercepts ranged from −0.49 to 0.50. Figure 1 shows the variation in predicted risks by the three risk calculations. Applying the three risk calculations for the prediction of PONV resulted in different predicted risks for the individual patients.

The risk calculation that included fixed and random effects (the conditional risk calculation) showed the best overall discriminative ability in the development data (Table 2, c-index 0.69). The discriminative ability of the standard model was similar to the discriminative ability of risks that were only based on the fixed effects of the random intercept model (the marginal risk calculation) (c-index both 0.66). The difference in discriminative ability among the standard model and marginal and conditional risk calculations disappeared when the c-index was estimated within each anesthesiologist, because the random anesthesiologist effects included in the conditional risk calculation only contributes to discrimination of patients treated by different anesthesiologist.

**Table 2:** **Apparent and test performance of the PONV models described in Table**1

	**Apparent performance**			**Test performance**	
	***Standard model***	***Marginal risk calculation***	***Conditional risk calculation***	***Standard model***	***Marginal risk calculation***
Harrell’s C-index †	0.66 (0.014)	0.66 (0.014)	0.69 (0.013)	0.68 (0.014)	0.67 (0.014)
C-index within clusters ‡	0.63 (0.089)	0.62 (0.097)	0.62 (0.097)	0.72 (0.129)	0.70 (0.127)
Calibration intercept*	0.00	0.01	0.00	0.13	0.14
Calibration intercept* within clusters ‡	0.01 (0.329)	-0.00 (0.385)	0.00 (0.000)	0.10 (0.339)	0.11 (0.380)
Calibration slope	1	0.95	1.08	1.08	0.99
Calibration slope within clusters ‡	0.97 (0.189)	0.94 (0.251)	1.08 (0.000)	1.06 (0.060)	1.00 (0.027)

† overall performance (standard error).

‡ within anesthesiologist performance (standard deviation).

* With calibration slope equal to 1 (i.e. calibration in the large).

The standard model showed excellent apparent calibration in the large, when estimated with the overall performance estimates (Table 2). Apparent calibration in the large (overall) for the marginal risk calculation was almost optimal. However, calibration in the large assessed within clusters showed that predicted and observed incidences differed for some anesthesiologist. The standard deviations of calibration in the large within clusters were 0.329 and 0.385 for respectively the standard model and the marginal risk calculation (Table 2). The differences in predicted and observed incidences within some anesthesiologists were slightly smaller for the standard model compared to the marginal risk calculation, because predictors included in the marginal risk calculation did not contain information of the anesthesiologist specific intercept as these predictors were adjusted for the random anesthesiologist effects. For the conditional risk calculation, the calibration in the large within clusters and corresponding standard deviation were close to 0, which means that the observed and predicted incidences were similar among all anesthesiologists. This is due to the inclusion of the random anesthesiologist effects in the risk calculation, which comprises an estimate of the cluster specific incidence.

The calibration slope assesses the correlation between predicted and observed risks for all patients (overall performance), or for patients treated by a particular anesthesiologist (within cluster performance). The overall and within cluster calibration slopes of the marginal risk calculation were slightly smaller compared to the calibration slopes of the standard model. The overall and within cluster calibration slopes of the conditional risk calculation were >1 in the development data (i.e. predicted risks lower than observed risks), because the anesthesiologist effects were shrunken to the average effect by the random intercept model. The standard deviations of the calibration slopes within clusters were limited for all models, indicating that the observed and predicted risks differed similarly among the anesthesiologists (Table 2).

The standard and the marginal risk calculation had similar test performance, which was estimated in patients treated by 19 other anesthesiologists (overall c-index resp. 0.68 and 0.67) (Table 2). The test performance as evaluated with the overall and within cluster c-indexes was even higher than in the apparent performance. Possible reasons are stronger true predictor effects in the test data, differences in case-mix and randomness [25]. (The models were not re-calibrated in the external data). The overall and within cluster calibration in the large were too high for both models in the external data, indicating that the predicted risks were lower than the observed proportions of PONV. The calibration slopes from the standard model were larger than slopes from the random intercept model, as was also shown in the apparent validation.

### Simulation study

The simulation study also showed similar overall discriminative ability for the standard model and the marginal risk calculation (apparent performance, c-index both 0.79, for ICC = 5%, Table 3). Discrimination of the conditional risk calculation was slightly better compared to the standard model and the marginal risk calculation (c-index 0.82, 2.5 and 97.5 percentiles 0.79; 0.84). The apparent calibration intercept and slope were ideal for standard model when assessing the overall performance, and ideal for the marginal risk calculation when assessing the within cluster performance. As in the empirical data, the overall and within cluster calibration slopes were too high for the conditional risk calculation due to shrinkage of the random center effects. Variation in the calibration in the large estimates within centers was lowest for the conditional risk calculation (calibration in the large and corresponding standard deviation both 0) (Table 3). The test performance of the standard model and the marginal risk calculation, as assessed in the source population, showed that the performance of these models was similar (Table 3). The difference between the apparent and test performance may be can be interpreted as optimism in model performance. Optimism in overall and within cluster performance was similar for the standard model and the marginal risk calculation. For instance, the difference in apparent and test calibration slopes (overall) was 0.04 for both models (Table 3). The risks for patients clustered within different centers was similar in these data (ICC 5%), which means that including center effects in the prediction model (i.e. random intercept model) cannot improve predictive performance considerably. Consequently, the performance of the models was similar, in the data with small differences in risks among centers.

**Table 3:** **Simulation results in a domain with ICC** = **5**%, **Pearson correlation X1 and random effect 0**.**0**

	**Apparent performance**			**Test performance**	
	***Standard model***	***Marginal risk calculation***	***Conditional risk calculation***	***Standard model***	***Marginal risk calculation***
Harrell’s C-index †	0.79 (0.766; 0.816)	0.79 (0.766; 0.816)	0.82 (0.788; 0.839)	0.78 (0.780; 0.785)	0.78 (0.780; 0.785)
C-index within clusters ‡	0.80 (0.077)	0.80 (0.077)	0.80 (0.077)	0.79 (0.031)	0.79 (0.031)
Calibration intercept*	0.00	0.02	0.00	0.04	0.07
Calibration intercept* within clusters ‡	-0.02 (0.442)	-0.00 (0.453)	0.00 (0.000)	0.01 (0.494)	0.05 (0.500)
Calibration slope	1.00	0.97	1.05	0.96	0.92
Calibration slope within clusters ‡	1.05 (0.090)	1.01 (0.092)	1.06 (0.000)	1.00 (0.022)	0.96 (0.021)

* With calibration slope equal to 1 (i.e. calibration in the large).

† overall performance (2.5 and 97.5 percentiles).

‡ median of overall performance from 100 simulations (median of within cluster performances).

The similarity in performance among the models disappeared when the ICC was 15% or 30% (Table 4, Additional file 1: Table S3). The discriminative ability of the conditional risk calculation was more accurate compared to the standard model and the marginal risk calculation. The apparent overall c-indexes and corresponding 2.5%; 97.5% range were respectively 0.85 (0.82; 0.87), 0.77 (0.74; 0.80) and 0.77 (0.73; 0.80) (Table 4). Assessment of the apparent performance of the standard model and the marginal risk calculation showed that the c-indexes remained similar in data with a higher ICC, however, the calibration parameters differed. The standard calibration in the large and calibration slope were equal to the line of identity for the standard model, but not for the marginal risk calculation. However, the calibration parameters assessed within clusters were on average more accurate for the marginal risk calculation (−0.00 and 1.00), compared to the standard model (−0.18 and 1.18). The evaluation of the standard model and the marginal risk calculation in the source population (test performance) showed similar results compared to the evaluation in the study sample (apparent performance) (Table 4).

**Table 4:** **Simulation results in a domain with ICC** = **15**%, **Pearson correlation X1 and random effect 0.0**

	**Apparent performance**			**Test performance**	
	***Standard model***	***Marginal risk calculation***	***Conditional risk calculation***	***Standard model***	***Marginal risk calculation***
Harrell’s C-index †	0.77 (0.735; 0.802)	0.77 (0.734; 0.801)	0.85 (0.818; 0.873)	0.77 (0.762; 0.769)	0.77 (0.763; 0.769)
C-index within clusters ‡	0.80 (0.085)	0.80 (0.086)	0.80 (0.086)	0.80 (0.037)	0.80 (0.037)
Calibration intercept*	0.00	0.19	0.00	-0.00	0.20
Calibration intercept* within clusters ‡	-0.18 (0.941)	-0.00 (1.005)	0.00 (0.000)	-0.19 (0.967)	0.01 (1.024)
Calibration slope	1.00	0.85	1.07	0.97	0.83
Calibration slope within clusters ‡	1.18 (0.096)	1.00 (0.082)	1.07 (0.000)	1.15 (0.011)	0.99 (0.008)

* With calibration slope equal to 1 (i.e. calibration in the large).

† overall performance (2.5 and 97.5 percentiles).

‡ median of overall performance from 100 simulations (median of within cluster performances).

Further, the apparent and test performance showed that, in data with an ICC of 15% or 30%, the standard deviations of the calibration in the large within clusters for the standard model and the marginal risk calculation were higher (e.g. respectively 0.94 and 1.01), compared to the standard deviations in data with an ICC of 5% (respectively 0.44 and 0.45) (Tables 4 and 3). So, when predictions were based on models neglecting center specific effects, the correlation between the observed and predicted incidences within centers differed among centers, especially in data with a high ICC. The (standard deviations of the) c-indexes within clusters were not influenced by a higher ICC.

Tables 5 and 6 show the results of simulations investigating the influence of the number of centers on model performance. Especially when the number of centers is low – e.g. 5 centers –, it is more difficult to estimate accurate random intercepts and corresponding center effects. This potentially affects the performance of the random intercept logistic regression model. However, as in Table 3, the performance of the standard model and the marginal risk calculation were similar, and the conditional risk calculation had the most accurate performance.

**Table 5:** **Simulation results in a domain with ICC** = **5**%, **Pearson correlation X1 and random effect 0**.**0**, **number of patients 100**, **number of centers 5**

	**Apparent performance**			**Test performance**	
	***Standard model***	***Marginal risk calculation***	***Conditional risk calculation***	***Standard model***	***Marginal risk calculation***
Harrell’s C-index †	0.82 (0.742; 0.889)	0.82 (0.743; 0.888)	0.84 (0.752; 0.913)	0.77 (0.718; 0.779)	0.77 (0.719; 0.779)
C-index within clusters ‡	0.82 (0.100)	0.82 (0.096)	0.82 (0.096)	0.77 (0.032)	0.77 (0.032)
Calibration intercept*	-0.00	0.00	0.01	0.02	0.06
Calibration intercept* within clusters ‡	-0.00 (0.295)	-0.00 (0.319)	0.01 (0.000)	-0.01 (0.537)	0.03 (0.545)
Calibration slope (overall)	1.00	0.99	1.05	0.71	0.69
Calibration slope within clusters ‡	1.06 (0.100)	1.01 (0.108)	1.07 (0.000)	0.74 (0.017)	0.72 (0.018)

* With calibration slope equal to 1 (i.e. calibration in the large).

† overall performance (2.5 and 97.5 percentiles).

‡ median of overall performance from 100 simulations (median of within cluster performances).

**Table 6:** **Simulation results in a domain with ICC** = **5**%, **Pearson correlation X1 and random effect 0**.**0**, **number of patients 1000**, **number of centers 50**

	**Apparent performance**			**Test performance**	
	***Standard model***	***Marginal risk calculation***	***Conditional risk calculation***	***Standard model***	***Marginal risk calculation***
Harrell’s C-index †	0.79 (0.762; 0.819)	0.79 (0.762; 0.819)	0.82 (0.787; 0.847)	0.78 (0.779; 0.785)	0.78 (0.779; 0.785)
C-index within clusters ‡	0.80 (0.123)	0.80 (0.123)	0.80 (0.123)	0.79 (0.031)	0.79 (0.031)
Calibration intercept*	0.00	0.03	0.01	-0.01	0.03
Calibration intercept* within clusters ‡	-0.03 (0.455)	-0.00 (0.468)	0.01 (0.000)	-0.03 (0.492)	0.00 (0.501)
Calibration slope (overall)	1.00	0.96	1.09	0.96	0.92
Calibration slope within clusters ‡	1.05 (0.095)	1.00 (0.096)	1.10 (0.000)	1.00 (0.027)	0.96 (0.026)

* With calibration slope equal to 1 (i.e. calibration in the large).

† overall performance (2.5 and 97.5 percentiles).

‡ median of overall performance from 100 simulations (median of within cluster performances).

The differences in performance between standard and random intercept models were smaller, when the clustering (i.e. the center effect) was associated with one of the predictors (Additional file 1: Tables S1, S2, and S4). For ICC values of 15% or 30%, the model performance of the standard model and the marginal risk calculation was better compared to model performance in datasets without associations between the clustering and a predictor. Finally, we compared the standard and random intercept model in data with a low incidence of the outcome Y (3%) (Additional file 1: Table S5). We sampled in total 1000 patients clustered in 20 centers. The performance of the models was similar. Only the calibration intercept of the marginal risk calculation showed an underestimation of the outcome incidence (intercept 0.14). The calibration intercept within clusters of the conditional risk calculation had a lower variance (0.00) compared to the other models.

## Discussion

We compared standard logistic regression with random intercept logistic regression for the development of clinical prediction models in clustered data. Our example with empirical data showed similar overall discriminative ability of the standard model and the random intercept model, if the cluster specific effects (estimated by a random intercept) were not used in the risk calculation. If the cluster specific effects of the random intercept model could be used for predictions, the overall discrimination and calibration in the large within clusters improved. This was confirmed in the simulation studies. The quality of model calibration depended on how the calibration was estimated. Standard developed prediction models showed better overall calibration than random intercept prediction models using the average of cluster effect, but the latter showed better calibration within clusters than standard developed models.

Predicted risks from the random intercept model were calculated using only the fixed predictor effects (the marginal risk calculation), or both fixed predictor effects and random cluster effects (the conditional risk calculation). The conditional risk calculation, including the same fixed predictor effects as the marginal risk calculation, showed the highest overall discrimination, apparently due to inclusion of the cluster specific information in the prediction model. The conditional risk calculation could only be applied and evaluated in the development data. To evaluate this risk calculation in subjects from new clusters, the new cluster effects should be known.

The differences that we found in calibration parameters between the standard model and random intercept logistic regression model (either used with the marginal or conditional risk calculation) slightly disappeared when the cluster effect was correlated with one of the predictors (Pearson correlation coefficient between cluster and X1 = 0.4, see Additional file 1: Table S1, S2 and S4). Especially the standard deviations of the calibration in the large within clusters were lower in data with correlation (e.g. 0.110 for the apparent performance of the standard model, ICC 5%) as compared to simulated data without correlation between the cluster effect and predictor X1 (standard deviation 0.444). So, the predicted and observed incidences within several clusters were better in agreement in data with the correlation. Due to the correlation of predictor and cluster, the predictor X1 contained information of the cluster and was able to predict (partly) the cluster specific incidence, hence improving the calibration in the large within clusters.

Until now, random effects models are mainly used to obtain correct estimates of intervention effects in cluster randomized trials [5,26], or causal effects in multilevel etiologic studies [27,28] and more recently in meta-analysis with individual patient data. The focus in such studies is on the effect estimate of one main factor, usually the intervention or exposure, which may be adjusted for confounding factors. In prediction studies, the focus is on all estimated predictor effects. All effects combined in the linear predictor results in an absolute risk estimate. Indeed, we found in our data, that the predictor effects for PONV were different in the random intercept logistic regression model compared to the standard model (Table 1). The different predictor effects, however, did not result in clear improvements in model performance (discrimination and calibration) between the marginal risk calculation and the standard model. This may be the result of relatively low clustering (ICC = 3.2%) in our empirical data. The simulations showed that particularly model calibration within clusters was better for the random intercept logistic regression models than the standard model, if the data were stronger clustered (ICC = 15%).

The differences in overall and within cluster performance measures – both discrimination and calibration measures – raise the question what estimates are preferable in the presence of clustered data. Measures that assess the within cluster performance – i.e. performance per cluster – will be probably more informative than overall measures, since prediction models are to be used by individual clinicians or treatment centers. Reporting the variability of the within cluster performance measures found in the original development data set, indicates future users whether the model performance will differ widely among centers. Wide differences would implicate that the model may need updating for individual centers with center specific information.

## Conclusion

In summary, we compared prediction models that were developed with random intercept or standard logistic regression analysis in clustered data. Adding the cluster effect in a prediction model increases the amount of predictive information, resulting in improved overall discriminative ability and calibration in the large within clusters. Particularly if cluster effects are relatively strong (ICC larger than 5%), prediction modeling with inclusion of the cluster effect in the model will result in better performance than models not including cluster specific effects.

## Abbreviations

PONV: Postoperative nausea and vomiting; c-index: Concordance index; ICC: Intraclass correlation coefficient

## Competing interests

All authors (W Bouwmeester, JWR Twisk, TH Kappen, WA van Klei, KGM Moons, Y Vergouwe) state that they have no conflict of interests.

## Authors’ contributions

Study design and analysis: WB, YV. Drafting manuscript: WB. Study design and reviewing the manuscript: JWRT, KGMM, THK, WAvK, YV. All authors read and approved the final manuscript.

## Pre-publication history

The pre-publication history for this paper can be accessed here:

http://www.biomedcentral.com/1471-2288/13/19/prepub

## Supplementary Material

Additional file 1: Table S1 Simulation results in a domain with ICC = 5%, Pearson correlation X1 and random effect 0.4. Apparent performance. **Table S2** Simulation results in a domain with ICC = 15%, Pearson correlation X1 and random effect 0.4. **Table S3** Simulation results in a domain with ICC = 30%, Pearson correlation X1 and random effect 0.0. **Table S4** Simulation results in a domain with ICC = 30%, Pearson correlation X1 and random effect 0.4. **Table S5** Simulation results in a domain with ICC = 5%, Pearson correlation X1 and random effect 0.0, outcome incidence 3% in 1000 patients. (DOC 83 kb) Click here for file
