# Supplementary material for: Prediction models for clustered data: comparison of a random intercept and standard regression model
Source: BMC Med Res Methodol. 2013 Feb 15;13:19. doi: 10.1186/1471-2288-13-19 (PMC3658967; doi:10.1186/1471-2288-13-19)
Supplement: Additional file 1: Table S1 — Simulation results in a domain with ICC = 5%, Pearson correlation X1 and random effect 0.4. Apparent performance. Table S2 Simulation results in a domain with ICC = 15%, Pearson correlation X1 and random effect 0.4. Table S3 Simulation results in a domain with ICC = 30%, Pearson correlation X1 and random effect 0.0. Table S4 Simulation results in a domain with ICC = 30%, Pearson correlation X1 and random effect 0.4. Table S5 Simulation results in a domain with ICC = 5%, Pearson correlation X1 and random effect 0.0, outcome incidence 3% in 1000 patients. (DOC 83 kb) [file 1471-2288-13-19-S1.doc]

**Additional file 1**

**Table S1** Simulation results in a domain with ICC = 5%, Pearson correlation X1 and random effect 0.4. Apparent performance.

|  | **Apparent performance** | | | **Test performance** | |
| --- | --- | --- | --- | --- | --- |
|  | *Standard model* | *Marginal risk calculation* | *Conditional risk calculation* | *Standard model* | *Marginal risk calculation* |
| Harrell’s C-index † | 0.78 (0.747; 0.806) | 0.78 (0.746; 0.806) | 0.79 (0.750; 0.815) | 0.775 (0.772; 0.778) | 0.775 (0.772; 0.778) |
| C-index within clusters ‡ | 0.77 (0.083) | 0.77 (0.083) | 0.77 (0.083) | 0.76 (0.031) | 0.76 (0.031) |
| Calibration intercept* | 0.00 | 0.00 | 0.00 | 0.01 | 0.02 |
| Calibration intercept* within clusters ‡ | -0.00 (0.110) | -0.00 (0.113) | 0.00 (0.000) | -0.01 (0.194) | -0.00 (0.196) |
| Calibration slope | 1.00 | 1.00 | 1.01 | 0.99 | 0.98 |
| Calibration slope within clusters ‡ | 1.00 (0.049) | 1.00 (0.050) | 1.02 (0.000) | 0.97 (0.003) | 0.97 (0.003) |

**Table S2** Simulation results in a domain with ICC = 15%, Pearson correlation X1 and random effect 0.4.

|  | **Apparent performance** | | | **Test performance** | |
| --- | --- | --- | --- | --- | --- |
|  | *Standard model* | *Marginal risk calculation* | *Conditional risk calculation* | *Standard model* | *Marginal risk calculation* |
| Harrell’s C-index † | 0.80 (0.778; 0.832) | 0.80 (0.778; 0.832) | 0.84 (0.803; 0.864) | 0.80 (0.796; 0.802) | 0.80 (0.794; 0.802) |
| C-index within clusters ‡ | 0.79 (0.079) | 0.79 (0.078) | 0.79 (0.078) | 0.79 (0.032) | 0.79 (0.032) |
| Calibration intercept* | 0.00 | 0.10 | 0.00 | -0.02 | 0.10 |
| Calibration intercept* within clusters ‡ | -0.09 (0.556) | -0.00 (0.587) | 0.00 (0.000) | -0.14 (0.610) | -0.02 (0.639) |
| Calibration slope | 1.00 | 1.01 | 1.06 | 0.97 | 0.99 |
| Calibration slope within clusters ‡ | 0.99 (0.077) | 1.00 (0.080) | 1.06 (0.000) | 0.92 (0.005) | 0.95 (0.005) |

**Table S3** Simulation results in a domain with ICC = 30%, Pearson correlation X1 and random effect 0.0

|  | **Apparent performance** | | | **Test performance** | |
| --- | --- | --- | --- | --- | --- |
|  | *Standard model* | *Marginal risk calculation* | *Conditional risk calculation* | *Standard model* | *Marginal risk calculation* |
| Harrell’s C-index † | 0.74 (0.697; 0.791) | 0.74 (0.695; 0.791) | 0.88 (0.843; 0.907) | 0.73 (0.727; 0.734) | 0.73 (0.729; 0.734) |
| C-index within clusters ‡ | 0.80 (0.093) | 0.80 (0.091) | 0.80 (0.091) | 0.79 (0.045) | 0.80 (0.045) |
| Calibration intercept* | 0.00 | 0.27 | 0.00 | 0.03 | 0.30 |
| Calibration intercept* within clusters ‡ | -0.25 (1.397) | -0.00 (1.551) | 0.00 (0.000) | -0.22 (1.401) | 0.04 (1.547) |
| Calibration slope | 1.00 | 0.73 | 1.07 | 0.97 | 0.71 |
| Calibration slope within clusters ‡ | 1.34 (0.114) | 1.01 (0.087) | 1.07 (0.000) | 1.34 (0.048) | 0.98 (0.033) |

**Table S4** Simulation results in a domain with ICC = 30%, Pearson correlation X1 and random effect 0.4.

|  | **Apparent performance** | | | **Test performance** | |
| --- | --- | --- | --- | --- | --- |
|  | *Standard model* | *Marginal risk calculation* | *Conditional risk calculation* | *Standard model* | *Marginal risk calculation* |
| Harrell’s C-index † | 0.82 (0.791; 0.847) | 0.82 (0.787; 0.842) | 0.88 (0.836; 0.915) | 0.82 (0.811; 0.816) | 0.81 (0.803; 0.816) |
| C-index within clusters ‡ | 0.80 (0.090) | 0.80 (0.090) | 0.80 (0.090) | 0.79 (0.036) | 0.80 (0.037) |
| Calibration intercept* | 0.00 | 0.20 | 0.00 | -0.05 | 0.19 |
| Calibration intercept* within clusters ‡ | -0.18 (1.016) | -0.00 (1.077) | 0.00 (0.000) | -0.28 (1.059) | -0.05 (1.114) |
| Calibration slope (overall) | 1.00 | 1.01 | 1.06 | 0.97 | 0.98 |
| Calibration slope within clusters ‡ | 0.98 (0.099) | 1.01 (0.099) | 1.06 (0.000) | 0.90 (0.006) | 0.93 (0.003) |

**Table S5** Simulation results in a domain with ICC = 5%, Pearson correlation X1 and random effect 0.0, outcome incidence 3% in 1000 patients.

|  | **Apparent performance** | | | **Test performance** | |
| --- | --- | --- | --- | --- | --- |
|  | *Standard model* | *Marginal risk calculation* | *Conditional risk calculation* | *Standard model* | *Marginal risk calculation* |
| Harrell’s C-index † | 0.84 (0.772; 0.897) | 0.84 (0.772; 0.898) | 0.86 (0.782; 0.926) | 0.81 (0.791; 0.821) | 0.81 (0.792; 0.821) |
| C-index within clusters ‡ | 0.85 (0.152) | 0.85 (0.151) | 0.85 (0.151) | 0.82 (0.087) | 0.82 (0.087) |
| Calibration intercept* | 0.00 | 0.01 | 0.01 | 0.02 | 0.14 |
| Calibration intercept* within clusters ‡ | -0.01 (0.446) | -0.00 (0.459) | 0.01 (0.000) | -0.06 (0.450) | 0.06 (0.456) |
| Calibration slope (overall) | 1.00 | 0.99 | 1.07 | 0.88 | 0.86 |
| Calibration slope within clusters ‡ | 1.00 (0.125) | 1.00 (0.119) | 1.09 (0.000) | 0.88 (0.072) | 0.86 (0.069) |

* With calibration slope equal to 1.

† overall performance (2.5 and 97.5 percentiles).

‡ median of within performances from 100 simulations (median of standard deviations in 100 simulations).
